# Partial heart transplantation for destructive infective endocarditis

**DOI:** 10.1007/s12055-024-01728-3

**Published:** 2024-04-06

**Authors:** Carlos A. Mestres, Eduard Quintana

**Affiliations:** 1 Department of Cardiothoracic Surgery and The Robert WM Frater Cardiovascular Research Institute, The University of the Free State, PO Box 339 (Internal Box G32), Bloemfontein, 9300 South Africa; 2 Department of Cardiovascular Surgery, Hospital Clinic, University of Barcelona, Barcelona, Spain

**Keywords:** Infective endocarditis, Bivalvular, Aortic abscess, Ventricular septal defect, Root replacement

## Abstract

Infective endocarditis frequently spreads beyond the valve tissue, especially in the aortic location. Invasive endocarditis may lead to abscess formation or fistula, with substantial tissue loss. Here, the case of a 31-year-old male patient with destructive aortic and pulmonary valve endocarditis and a subaortic mural defect who underwent patch closure of the ventricular septal defect and aortic and pulmonary root replacement and right coronary artery bypass graft is presented. This is an uncommon condition and stress is placed on imaging of the technical aspects of the case.

## Introduction

A proportion of patients treated for infective endocarditis (IE) develops extravalvular extension of the disease, which may evolve into abscess formation or even fistulae towards different locations [1]. This entails more complex surgical procedures and, therefore, higher surgical risk eventually impacting perioperative outcomes. Complex procedures requiring reconstructions represent a challenge for the surgical team. It is agreed that a paradigmatic example of a difficult procedure in IE is the reconstruction of the aorta-mitral curtain when the fibrous skeleton of the heart is affected by the spread of the infection posteriorly, which is termed in different ways, with the “Commando operation” becoming nowadays popular [2]. The infection may also extend anteriorly beyond the valve leaflets reaching the interventricular septum and eventually advancing towards the pulmonary root leading to the need for other surgical options that have been seldomly reported.

Surgery for complex IE deals with a heterogeneity of cases, which precludes standardization of repair techniques across the spectrum of valve involvement. A variety of homologous, autologous, or prosthetic materials has been used for valve reconstruction, but outcomes in the acute phase are usually related to the preoperative patient condition and clinical characteristics rather than valve choice [3]. Specific situations that may fall within the category of case reports are treated according to the experience and expertise of a given surgeon or surgical team. In these situations, there may be no detailed preoperative planning; thus, surgical imagination and adaptation play a critical role in decisions made at the operating table. In extreme cases, heart transplantation has been indicated and performed [4].

A case of complex multivalvular IE was treated with the closure of a ventricular septal defect, and double root homotransplantation and coronary bypass could be currently interpreted as partial heart transplantation, bridging the differences, with recent experiences in the pediatric and adult population [5, 6]. This report deals with the technical aspects of a procedure that is not frequently reported in the setting of IE in the adult.

## Case description

A 31-year-old male, originally from Bolivia, had a history of non-screened cardiac murmur and relapsing dental infections, the latest 6 months before admission. He was admitted because of prolonged fever, shaking chills, and constitutional syndrome. He was treated for 10 days with oral levofloxacin because suspected urinary tract infection. Two days before admission, the patient reported pain in the right back.

Physical examination at the Emergency Room confirmed the following: blood pressure, 117/62 mmHg; electrocardiogram, normal sinus 94 bpm; axillary temperature, 37.9 °C; respiratory rate, 20 rpm; baseline oxygen saturation, 96%; pallor and stress; oral septic foci. Palpable peripheral pulses. On auscultation, there was an II/VI systolic murmur in all four major auscultation areas and no clicking, bubbling, or rattling sounds in the lungs. There was no peripheral edema but a 2-cm liver enlargement and no splenomegaly. Neurologically intact. The lower right back was painful on palpation.

Laboratory examinations yielded the following results: C-reactive protein 21.8 mg/dL; glucose 142 mg/dL; total bilirubin 0.6 mg/dL; amylase 77 mg/dL; Na^+^/K^+^ 134/3.9 mEq/L; white blood cell count 12,800 (N 78%; L 15%; M 6%; E 1%; B 0%); hemoglobin 89 g/L; hematocrit value 28%; platelets 184,000/mm^3^; prothrombin time 76%. Brucella, *Salmonella typhi*, Epstein-Barr virus, and hepatitis B and C virus serologies were negative. Blood cultures from peripheral veins (4/4) on admission day grew *Streptococcus mitis* sensitive to penicillin and erythromycin. The patient was initially placed on intravenous ceftriaxone 2 g/24 h, gentamicin 120 mg/12 h, and ampicillin 2 g/6 h.

On plain chest X-ray, there were signs of pulmonary congestion. Admission transthoracic echocardiography (TTE) suggested IE with involvement of the aortic and pulmonary valves and a pattern of periaortic abscess with multiple vegetations on the right sinus of Valsalva and a communication between the aorta and the right ventricle. There were also multiple highly mobile masses on the right ventricular outflow tract (RVOT) up to 20 × 5 mm. There was moderate-to-severe aortic regurgitation (AR) and moderate pulmonary insufficiency (PI) on a non-rheumatic valve. Left ventricular (LV) contractility fell within the normal range, and there were signs of right ventricular overload. The mitral and tricuspid valves were found to be anatomically and functionally normal.

Transoesophageal echocardiography (TOE) the following day yielded the same findings of the right sinus of Valsalva abscess and a fistula between the aorta and the right ventricle. There were RVOT mobile masses up to 20 × 5 mm and a prominent pulmonary valve (PV) vegetation. There was severe AR on a trileaflet valve. The mitral and tricuspid valves were anatomically and functionally normal with no vegetations.

### Surgery

With a strong indication for surgery because of acute bivalvular regurgitation and echocardiographic evidence of cavitary fistula (Class IB), the patient was operated on an emergency basis within 48 h from admission.

**Fig. 1:**
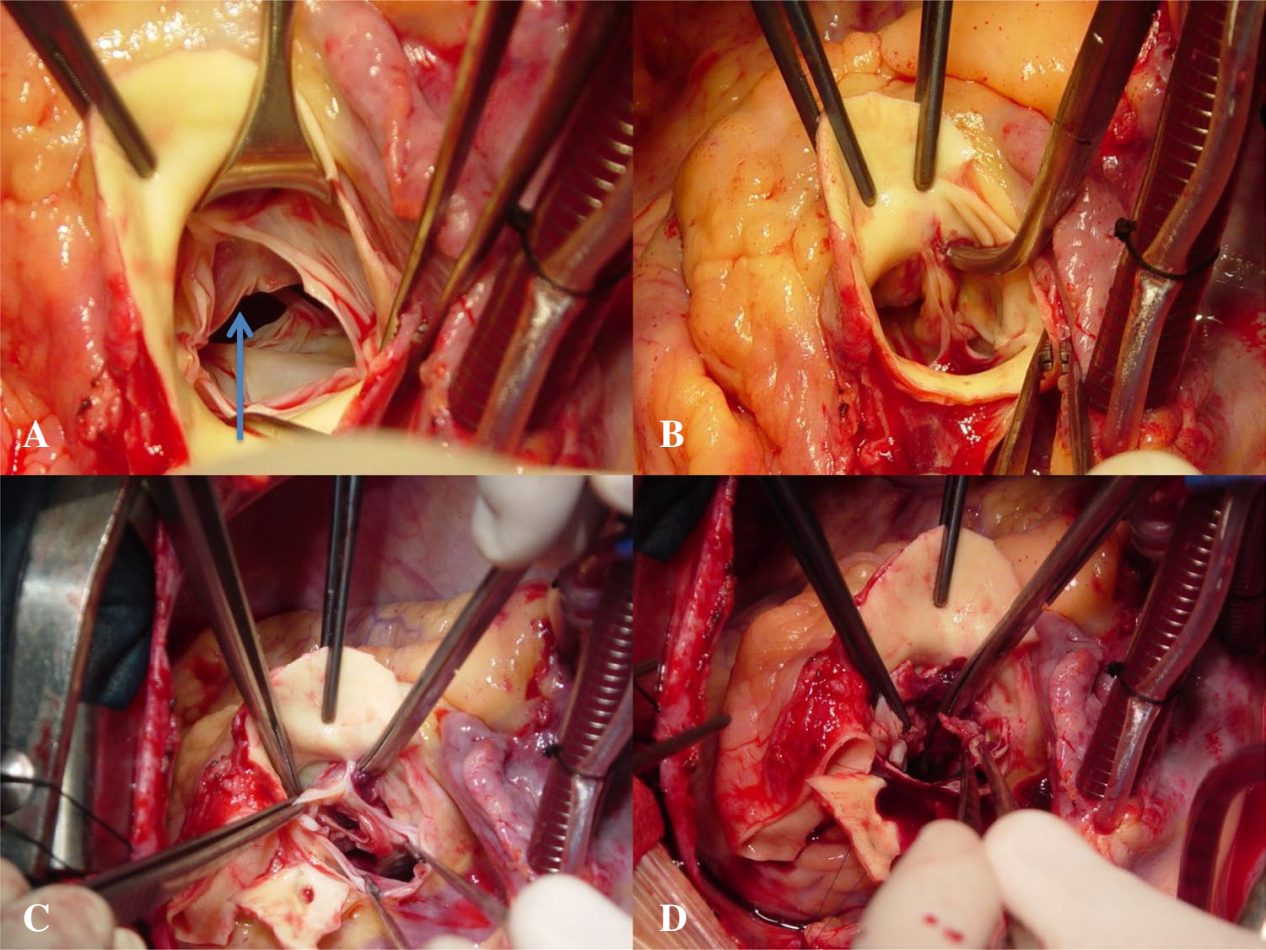
**A** Trileaflet aortic valve. Subaortic abscess (arrow). **B** Crile forceps introduced into the abscess cavity. **C** Subaortic abscess exposed after removal of the aortic root tissue. **D** Thrombotic abscess material concealing the septal defect

**Fig. 2:**
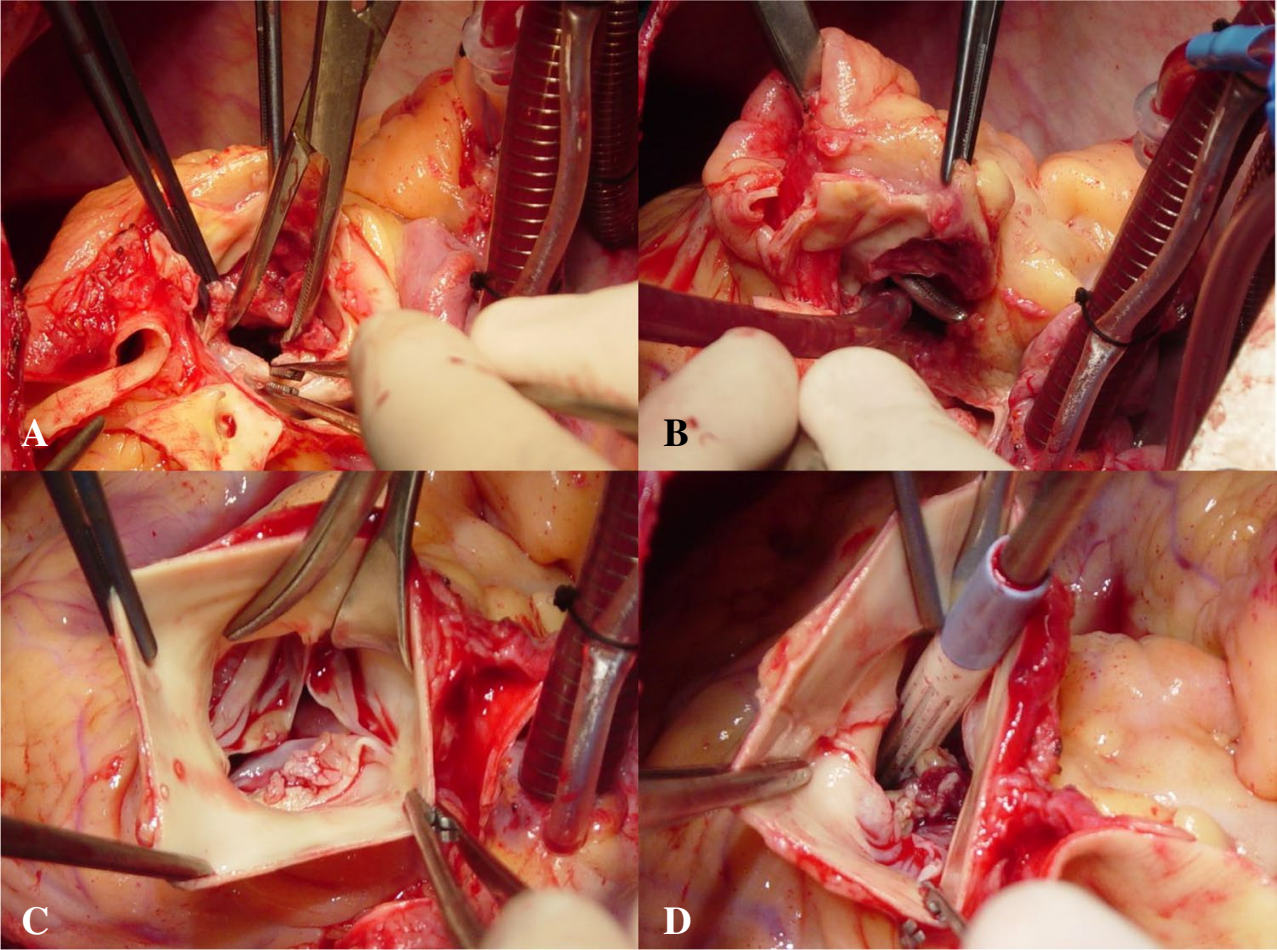
**A** Ventricular septal defect (20 mm). **B** Ventricular septal defect accessed through the transected pulmonary artery. **C** Pulmonary valve vegetations. **D** Right ventricle mural vegetations

Intraoperative findings were those of severe aortopulmonary IE with biventricular dilatation. There was an aortic abscess (40 × 30 mm) (Fig. 1A, B) circumferentially extending into the midventricular level (Fig. 1C, D) leading to an actual ventricular septal defect of about 20 mm in diameter, which was evident after removing the inflammatory thrombus occupying the abscess (Fig. 2A, B). The aortic valve (AV) was tricuspid, and there were vegetations on two PV leaflets. There was mural IE of the right ventricle (RV) with multiple masses (Fig. 2C, D). The abscess also involved the origin of the right coronary artery, which was detached from the root.

**Fig. 3:**
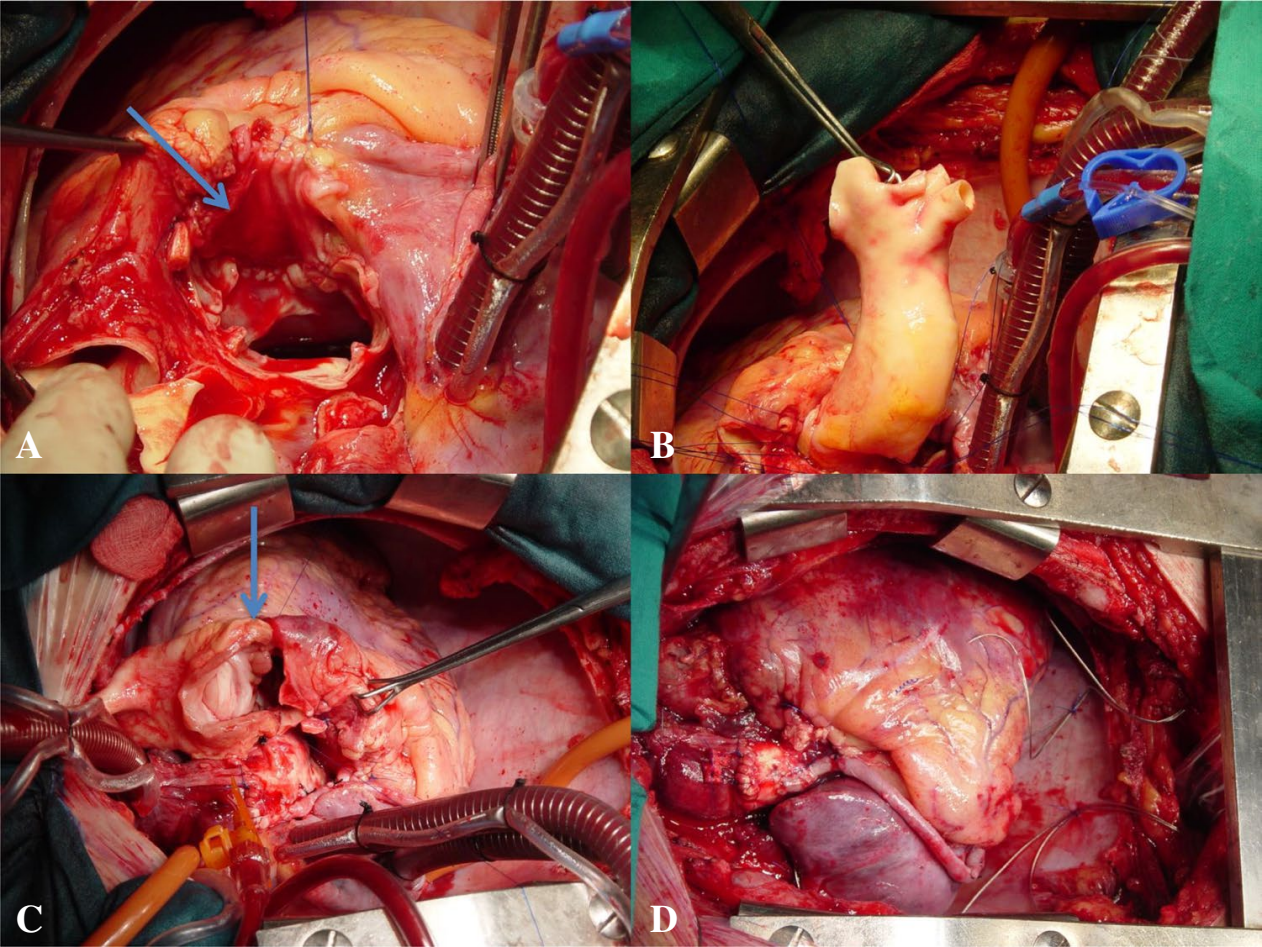
**A** Septal defect (arrow) and pericardial patch. **B** Homograft aortic root replacement. **C** Pulmonary root replacement. Proximal right ventricular suture line (arrow). **D** The completed operation. Double root replacement and a saphenous vein-to-distal right coronary artery

Cardiopulmonary bypass (CPB) was established with bicaval cannulation and aortic return. Myocardial protection was achieved with antegrade and continuous retrograde blood cardioplegia. The patient underwent closure of the ventricular septal defect with an autologous pericardial patch, which was sutured with a running 3/0 polypropylene suture (Fig. 3A). The aortic root was replaced with a 20–22-mm cryopreserved aortic homograft in which proximal and distal connections were performed with a running 4/0 polypropylene suture (Fig. 3B). The pulmonary root was replaced with a 27-mm cryopreserved pulmonary homograft (Fig. 3C). The right coronary artery was bypassed with a reversed saphenous vein graft (Fig. 3D). Aortic cross-clamping and CPB times were 104 and 166 min, respectively. The patient was successfully weaned off CPB with minimal dobutamine support.

His postoperative course was complicated with intubation-related dysphonia. Pre-discharge TTE disclosed normal functioning aortic and pulmonary homografts, a left ventricular ejection fraction of 60% with no ventricular septal leakage. In retrospective analysis, we were not able to determine whether the infection could have settled on a congenital ventricular septal defect (VSD) due to the characteristics of the preoperative echocardiography (TTE and TOE) or the intraoperative findings with regional destruction. However, the patient, as stated at the beginning of the description, had a history of a non-screened heart murmur, which could have suggested a congenital defect. We are only speculative here.

He was treated with ceftriaxone 1 g/24 h for 4 weeks and was discharged home on the 32nd postoperative day. The preoperative microbiological diagnosis was that of *S. mitis* IE that was confirmed by postoperative histology. In the histopathological study, reactive changes were detected in the adventitial tissue and mesothelial lining of the ascending aorta and main pulmonary artery. Acute inflammatory changes of severe intensity compatible with infective endocarditis were detected in the aortic and pulmonary valves due to the presence of gram-positive cocci, which were later confirmed as *S. mitis*.

One-year follow-up blood cultures were negative. He was followed at the outpatient clinic and was lost for follow-up exactly 2 years and 4 days after being discharged.

## Discussion

In this case, a double root replacement, closure of a true VSD, and a coronary bypass were performed. This is a complex surgical procedure in the setting of extensive tissue destruction due to invasive IE. Whichever terminology is used is irrelevant as what counts is the preoperative condition of the patient, the intraoperative conduct, and the outcome of the patient, who survived until he was lost for follow-up as he moved back to another location.

The recently coined terminology of partial heart transplantation [5] refers to the use of homovital anatomical structures directly taken from an organ donation. In the strict sense and for over six decades now, homograft (allograft) valve replacement is classically and universally considered tissue transplantation; most of these tissues are currently harvested from organ donors and are cryopreserved. On the other hand, the term homovital is an old one as homovital valve and vascular transplants have been performed in the past [7, 8]. The recently described operations in infants [5] obviously represent a step forward in the treatment of specific patients who cannot be treated with orthotopic heart transplantation.

This case appropriately illustrates the way IE destroys anatomical structures leading to severe hemodynamic disturbances and existent surgically options for organ function restoration. The so-called *Commando* operation [2] and its variants, orthotopic heart transplantation, and other complex techniques described in the past [9, 10] express the imagination of the surgeon to deal with conditions faced dismal prognosis in the past.

## Conclusion

The goal of surgery in complex cases of IE is, in association with a culture-directed antibiotic therapy, the restoration of cardiac function and infection eradication.

## Data Availability

Data on this case are available upon reasonable request.
